# UAV imaging and deep learning based method for predicting residual film in cotton field plough layer

**DOI:** 10.3389/fpls.2022.1010474

**Published:** 2022-10-06

**Authors:** Fasong Qiu, Zhiqiang Zhai, Yulin Li, Jiankang Yang, Haiyuan Wang, Ruoyu Zhang

**Affiliations:** ^1^ College of Mechanical and Electrical Engineering, Shihezi University, Shihezi, China; ^2^ Key Laboratory of Northwest Agricultural Equipment, Ministry of Agriculture and Rural Affairs, Shihezi, China

**Keywords:** cotton fields, plough layer, residual film pollution, UAV imaging, deep learning

## Abstract

In this paper, a method for predicting residual film content in the cotton field plough layer based on UAV imaging and deep learning was proposed to solve the issues of high labour intensity, low efficiency, and high cost of traditional methods for residual film content monitoring. Images of residual film on soil surface in the cotton field were collected by UAV, and residual film content in the plough layer was obtained by manual sampling. Based on the three deep learning frameworks of LinkNet, FCN, and DeepLabv3, a model for segmenting residual film from the cotton field image was built. After comparing the segmentation results, DeepLabv3 was determined to be the best model for segmenting residual film, and then the area of residual film was obtained. In addition, a linear regression prediction model between the residual film coverage area on the cotton field surface and the residual film content in the plough layer was built. The results showed that the correlation coefficient (R^2^), root mean square error, and average relative error of the prediction of residual film content in the plough layer were 0.83, 0.48, and 11.06%, respectively. It indicates that a quick and accurate prediction of residual film content in the cotton field plough layer can be realized based on UAV imaging and deep learning. This study provides certain technical support for monitoring and evaluating residual film pollution in the cotton field plough layer.

## 1 Introduction

Agricultural mulch has been widely used to increase soil temperature and moisture, suppress pests and weeds and reduce soil salinity which are conducive to improving crop yields and increase farmers’ incomes (Wang et al., 2020; Li et al., 2021). Plastic film mulching was introduced to China in the late 1970s. After decades of development, it has been widely used for the cultivation of cotton, corn, pepper, and other crops in northwest China, especially Xinjiang Province. It has made important contributions to the increases in the production and income levels of farmers in arid regions (Yan et al., 2006; Hu et al., 2019).

However, the “white pollution” caused by the widespread use of mulch is becoming increasingly prominent (Liu et al., 2014; Zhang et al., 2021; Li et al., 2021). As an economically important crop, cotton is mainly planted with film mulching in Xinjiang, China. Due to the continuous use of plastic mulch in cotton fields, and the incomplete recovery of the residual film, the amount of residual film in cotton field is as many as 42 ~540 kg/hm^2^, and the average residual amount exceeds 200 kg/hm^2^ (Wang, 1998; Xu et al., 2005; Zhao et al., 2017). However, the pollution of residual mulch film in cotton fields has a massive impact on cotton production (Zhang et al., 2016). For example, a large amount of plastic film left in the plough layer blocks the migration of water and nutrients, destroy the soil structure, and suppresses the germination of seeds and the growth of crop roots. In addition, the residual film is mixed into the seed cotton, which reduces the weight of the cotton (Zhang et al., 2020; Zong et al., 2021).

Fast and accurate monitoring of residual film pollution in farmlands has great significance for the control of residual film pollution. The assessment of the residual film pollution degree in farmlands is mainly through manual sampling. Zhang et al. (2017) adopted the manual stratified sampling method and arranged 7 sampling points at each monitoring site. The data analysis showed that the amount of residual film had an increasing trend of increasing. Wang et al. (2018) took layered samples of soil in cotton fields with different mulching years to analyse the areas and net weights of the residual film, and found that with the increase in years of mulching, the content of residual film increased yearly, with an average increase of approximately 10%. Besides, the residual film fragmented gradually and moved down to deep soil layer during ploughing. Qi et al. (2001) found that the residual film in the soil of cultivated land was mainly distributed in the plough layer (0~10 cm) accounting for approximately 2/3 of the total residual film. The rest was distributed in the 10~30 cm soil layer, and no residual film was found below 40 cm. However, the manual sampling method for residual film pollution monitoring in these studies is labor-intensive and inefficient and cannot meet the demands of rapid and accurate monitoring of residual film pollution.

UAV remote sensing technology has many advantages such as high operation efficiency, good mobility, low cost, and high spatial resolution (Cao et al., 2021). In recent years, it has been combined with technologies such as artificial intelligence and the Internet of Things and is widely used in agriculture for disease and pest prevention and control, sowing, and so on (Li et al., 2021; Su et al., 2021). In terms of monitoring residual film in farmland, some scholars have also preliminarily explored the application potential of the UAV remote sensing and identification methods. For example, Sun et al. (2018) used a six-rotor UAV equipped with a Sony NEX-5k camera for aerial photography and proposed an end-to-end method for identifying greenhouses and mulched farmland from drone images. As a result, the average accuracy achieved for the testing area was 97%. Zhu et al. (2019) used the images taken by drones of a research area and a fusion-based supervised image classification algorithm, and found that the recognition accuracy was 94.84%. Ning et al. (2021) proposed an improved deep semantic segmentation model based on the DeepLabv3+ network for plastic film identification and found that this method could effectively segment plastic film from farmland in UAV multispectral remote sensing images and the identification accuracy was 7.1% high than that of the visible light method. In conclusion, the rapid detection of residual film in farmland can be realized based on UAV remote sensing imaging technology. However, in fact, the residual film is mainly concentrated in the plough layer. Currently, there are few reports about detecting residual film pollution in the plough layer with the UAV images and deep learning method, and there is a lack of methods for the rapid detection of residual film pollution in the plough layer.

Therefore, in this study, the cotton field before spring sowing was taken as the research object, and a method for predicting residual film in the cotton field plough layer based on UAV imaging and deep learning was proposed. Through the identification of residual film on soil surface and the linear fitting with the actual weight data of residual film in the plough layer, the content of the residual film in the cotton field plough layer was detected rapidly and accurately. This study will provide a theoretical basis for further study of the rapid and accurate assessment technology residual film pollution in the plough layer and equipment development.

## 2 Materials and methods

### 2.1 Image data collection

**Figure 1:**
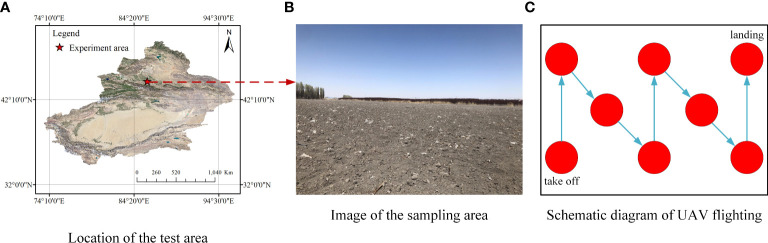
Test area and image acquisition. **(A)** Location of the test area; **(B)** Image of the sampling area; **(C)** Schematic diagram of UAV flighting.

The data was collected in Shihezi City, Xinjiang, China (42°10′~45°21′N, 84°20′~86°55′E, a.s.l. 450.8 m), where film mulching has been continuously used for cotton cultivation for many years. The data was collected before sowing in early April 2021 and a large amount of residual plastic film remained on the soil surface (**Figure 1**). A total of 30 cotton fields were selected. The images were collected in sunny and cloudy days, to increase the robustness of the subsequent model and enhance the adaptability to lighting. First, images were collected by drones, with a height of 5 m, and a total of 900 images, which constituted the training set for surface residual film recognition, were collected. Then, ten points (1 m^2^ per point) were selected to manually collect the residual film in the plough layer (0-10 cm in depth) in each cotton field (a total of 300 sampling points). All the residual film sampled were put into label bags.

**Figure 2:**
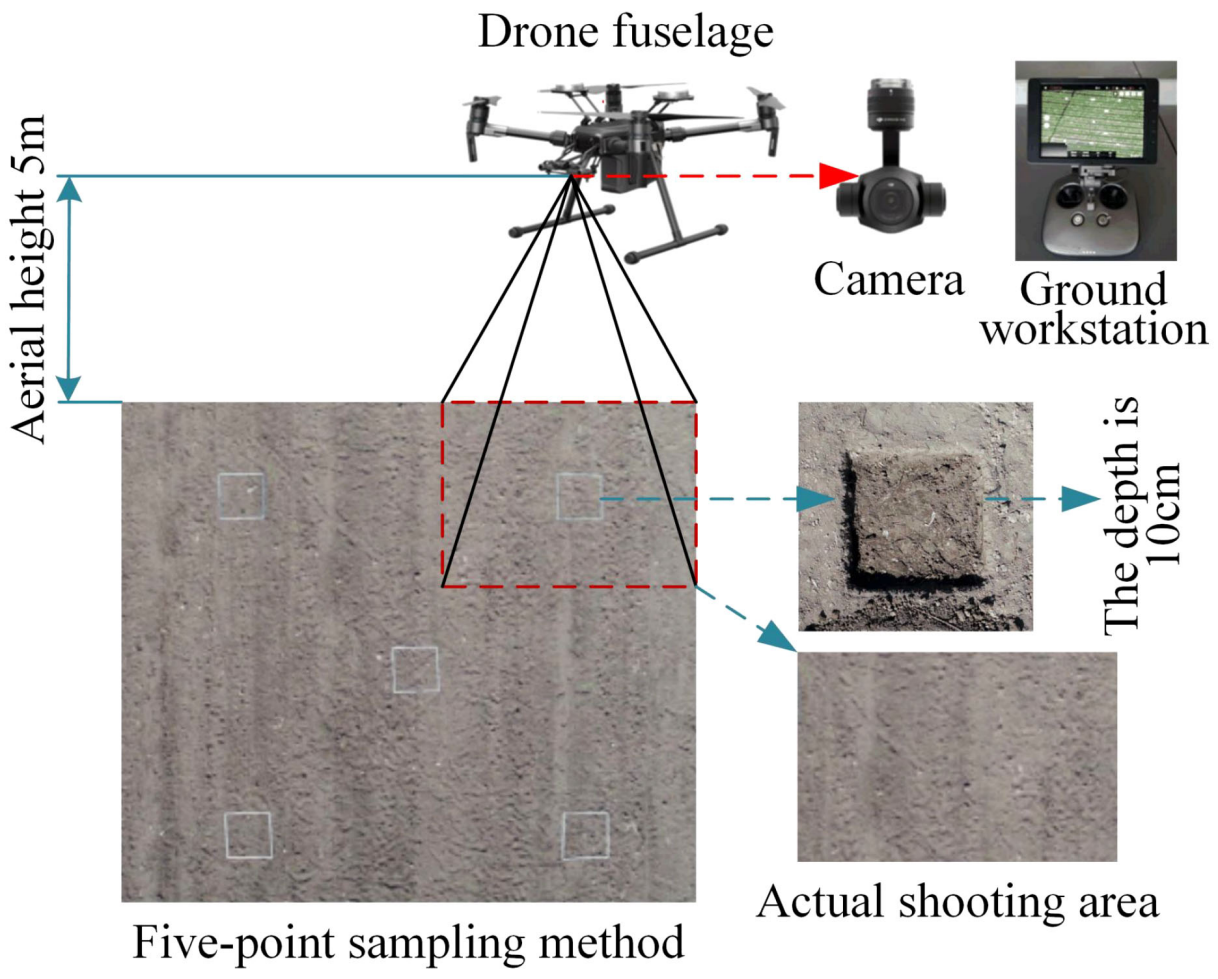
Schematic diagram of the data acquisition process.

The images of the cotton field were taken by a DJI M200 remote-controlled rotary-wing quadrotor drone (DJI MATRICE 200 V1, DJI, China) equipped with a ZENMUSE X4S cloud platform camera (FC6510, DJI, China). The camera had a fixed focal length of 8.8 mm, a F/2.8-11 focal ratio, and a field of view (FOV) of 84°. The resolution of the image was 5472×3078 pixels (JPG format). A DJI ground workstation (DJI, Shenzhen, China) was used to control the flight of the drone and transmit the images. After the drone landed, the images obtained were transferred to a laptop in the JPG format and checked for integrity. The sampling tools used for the collection of the residual film in the plough layer included a 1 m × 1 m folding ruler, a shovel, and a canvas. The equipment configuration and data acquisition process are shown in **Figure 2**.

### 2.2 Dataset construction and data augmentation

**Figure 3:**
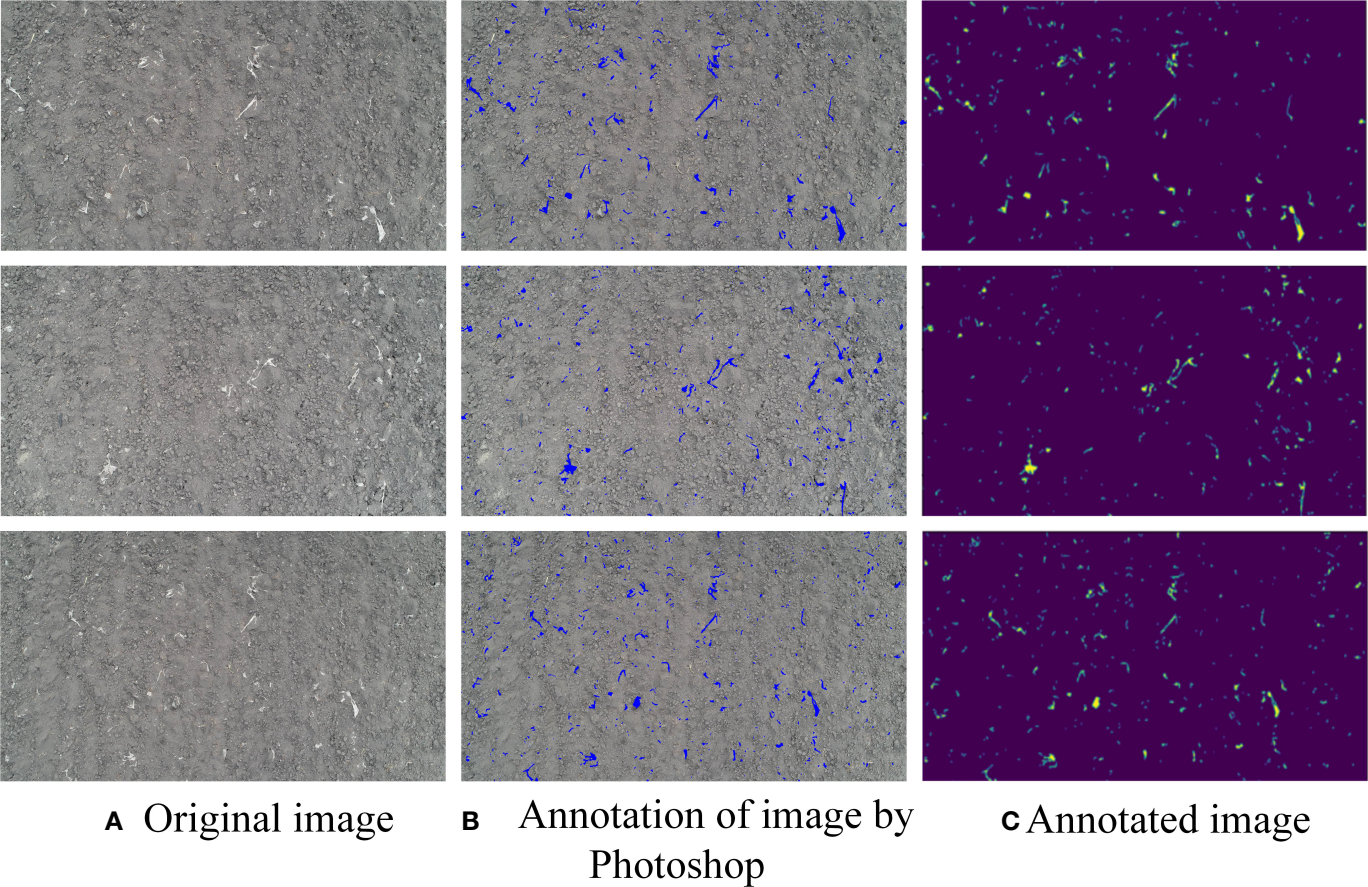
Schematic diagram of data annotation. **(A)** Original image; **(B)** Annotation of image by Photoshop; **(C)** Annotated image.

Due to the adhesion of soil on the surface of the residual film, the residual straw, and the drip irrigation belts, it was difficult to identify the residual film through a simple image processing method. In this study, a deep learning-based semantic segmentation method was used to identify the residual film. Deep learning is a new research direction in the field of machine learning. Semantic image segmentation is a very important research direction in computer vision. It identifies images at the pixel level, and its accuracy and efficiency greatly surpass those of other methods. In this study, the residual film in a total of 900 images of were manually marked with Photoshop CS5, and saved in the PNG format. The marked PNG images were used as a (**Figure 3**).

The marked dataset was enhanced by random cropping, that is, the image size was adjusted and the images were randomly cropped into images of the same size. Each raw image was normalized, and the corresponding marked image was randomly flipped. Then, the flipped images were normalized.

### 2.3 Semantic segmentation model construction

#### 2.3.1 LinkNet model

**Figure 4:**
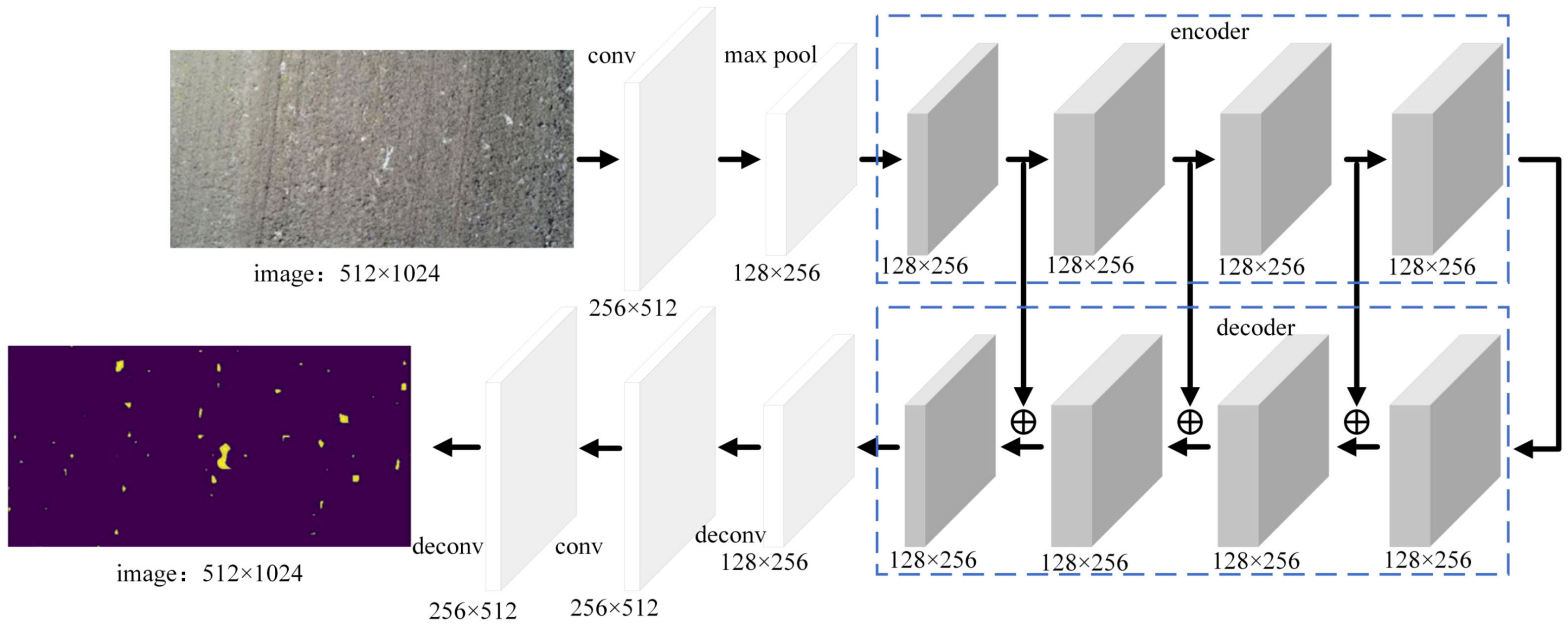
Schematic diagram of the LinkNet structure.

LinkNet uses the idea of a self-encoder with an architecture that includes two parts: an encoder and a decoder (**Figure 4**). The input contains 2 convolution layers and 1 pooling layer, the output contains 2 deconvolution layers, and the middle part contains 4 encoding layers and 4 decoding layers. The kernel size is 7×7, the number of kernels is 64, and the stride size is 2. The pooling layer utilizes the maximum pooling method. The maximum pooling window is 3 × 3, and the stride is 2. The upper part is the encoder structure, which contains 4 convolutional layers, and the encoder module performs forward propagation. The first 2 convolutional layers scale the input images, and the sizes of the images remain unchanged in the latter 2 convolutional layers. The output obtained by adding the outputs of the first 2 convolutional layers and the outputs of the latter 2 convolutional layers enters the decoder module. The decoder module in the lower part contains 2 convolutional layers and 1 deconvolutional layer; this module is equivalent to the back-propagation process and enlarges the images. After passing through the decoder module, the images enter the upsampling module. Then the images enter the second convolutional layer, and finally, the images are upsampled for the second time to obtain the final output images.

#### 2.3.2 FCN model

**Figure 5:**
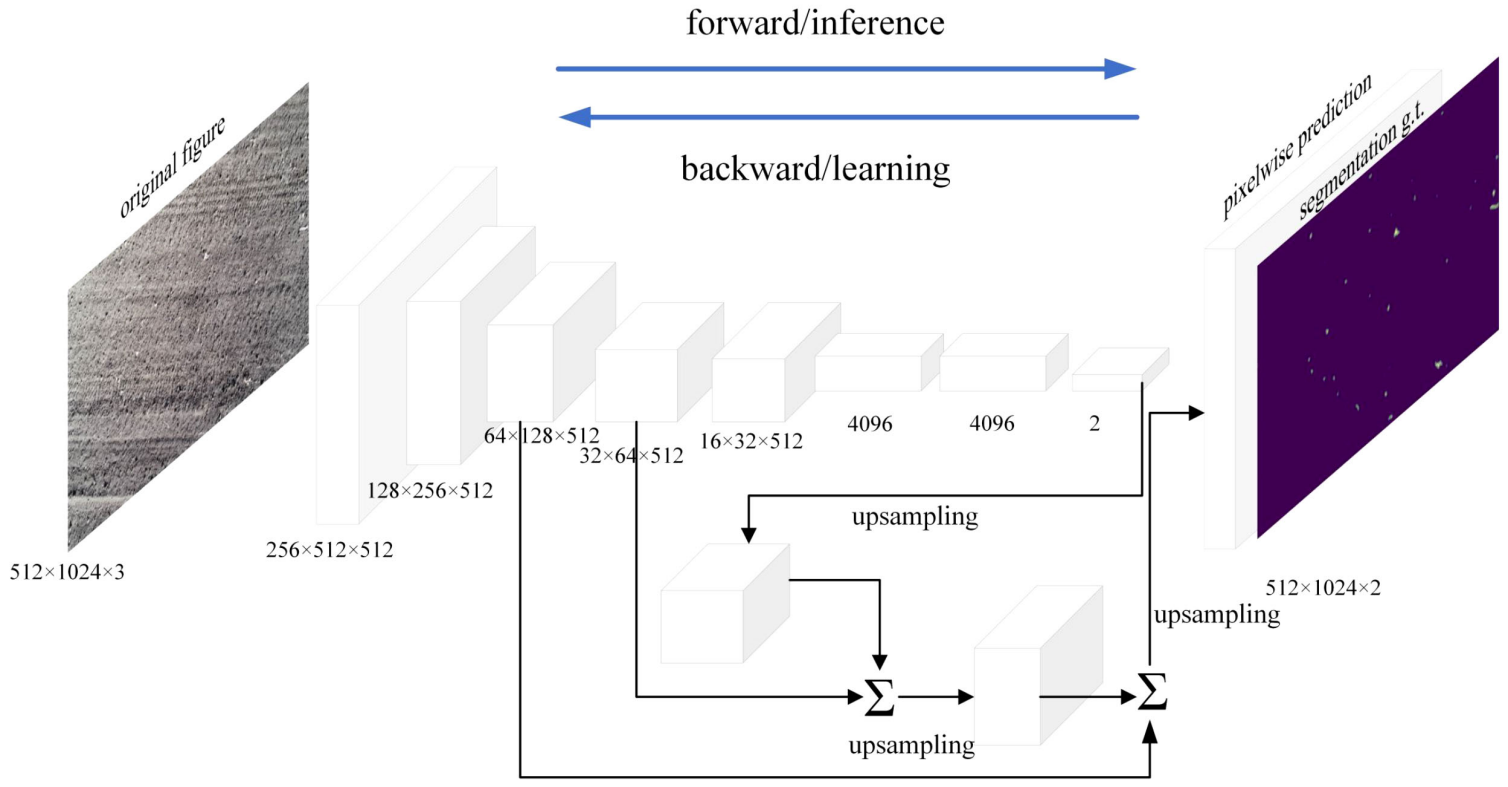
Schematic diagram of the FCN structure.

A Fully Convolutional Network (FCN) consists of two parts, full convolution and deconvolution layers (**Figure 5**). By referring to the Visual Geometry Group 16 (VGG16) pretraining network structure, pretraining weights were introduced in this study, and the fully connected layer of the VGG16 network was replaced with a 1 × 1 convolutional layer to solve the disadvantage that the number of neurons in the fully connected layer must be fixed and then to achieve input images of any size. The input convolution kernel size was 512, the convolution kernel size was 3 × 3, the input image size was 512 × 1024 pixels, and the number of channels was 3. By creating a submodel and obtaining the output of the middle layer of the VGG16 network, this study sets the last layer of the submodel as pool1 for upsampling, used the rectified linear unit (ReLU) function for activation, and then performed a convolution operation. Pool1 was added to the middle layer to obtain pool2, and in the same way, pool2 was upsampled, and another convolution operation were performed. Then, pool2 was added to the middle layer to obtain pool3. Pool3 was upsampled, and then convolution was performed again. The middle layers were added to obtain pool4, after completing the hopping structure, pool4 was upsampled to obtain the output images. The outputs were upsampled to obtain the final predicted images with the same size as that of the input image. The number of channels was 2. The model was created.

#### 2.3.3 DeepLabv3 model

**Figure 6:**
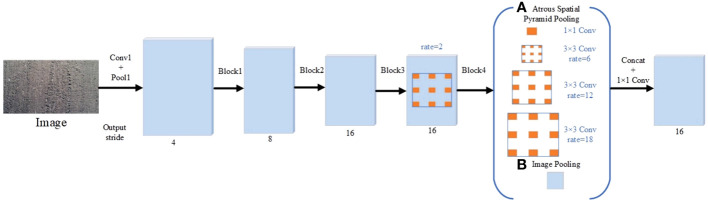
Schematic diagram of the DeepLabv3 structure. **(A)** Atrous Spatial Pyramid Pooling; **(B)** Image Pooling.

DeepLabv3, a multiscale image segmentation network based on the ResNet structure, is designed with serial and parallel atrous convolution module. It uses a variety of different convolution modules to obtain multiscale content information. It involves the hole convolution and the atrous spatial pyramid pooling (ASPP) with atrous convolution. The first three modules use the original convolution module, and the fourth module uses the atrous convolution module. The multiple-network atrous convolution expansion rate of the atrous convolution module is (2, 4, 8), the output stride is 16, and the size of the feature map is 32×32. Furthermore, the ASPP structure contains 4 parallel dilated convolutions, including one 1×1 convolution and three 3×3 convolutions. The ASPP structure obtains the global context information through a global average pooling layer and uses a 1×1 convolution to achieve fusion of the branch-processed features (**Figure 6**).

### 2.4 Model training

The proposed deep learning model was built based on Python 3.7 and the Jupyter Notebook editor using the Windows 10 desktop operating system running on an Intel(R) Gold 6126 CPU with a default frequency of 2.60 GHZ and 64 GB of memory. The graphics card used was an NVIDIA GeForce RTXTM 2060 (6 GB of video memory), and the model training framework adopted the TensorFlow 2.0 GPU version. In the experiment, 80% of the image samples were randomly selected as the training set, and the remaining were used as the validation set to verify the identification accuracy of each model. To improve the accuracy of the models, the Adam optimizer was used to optimize the three deep learning models. The learning rate was set to 0.001, the number of iterations was set to 50 epochs, the attenuation coefficient in the Adam optimizer was set to 0.9, and the loss function was the cross entropy loss function.

### 2.5 Evaluation indicators

In this study, five indicators, including accuracy precision, the mean intersection over union (MIOU), recall, precision, and F1-score, were used to evaluate the identification accuracy of the models. Accuracy is the proportion of positive samples predicted by the model to the total samples (Formula 1). Precision is the proportion of true positive samples predicted by the model to positive samples (Formula 2). Recall is the proportion of samples with predicted true values out of all true values (Formula 3). The F1-score is the harmonic mean of precision and recall (Formula 4). The MIOU is the mean of all categories of IOUs (Formula 5).


(1) $$Accuracy=\frac{TP+TN}{TP+FP+TN+FN}\times 100\%$$

(2) $$Precision=\frac{TP}{TP+FP}\times 100\%$$

(3) $$Recall=\frac{TP}{TP+FN}\times 100\%$$

(4) $$F1-Score=\frac{TP}{TP+FN}\times 100\%$$

(5) $$MIOU=\frac{1}{K+1}\sum_{i=0}^{k}\frac{TP}{TP+FP+FN}\times 100\%$$

Where TP denotes the number of correctly classified residual film pixels, FP denotes the number of background pixels that are misclassified as residual film pixels, FN denotes the number of residual film pixels that are incorrectly classified as background pixels, TN represents the number of correctly classified background pixels, and k is the total number of segmented residual film images.

### 2.6 Residual film coverage area detection method

**Figure 7:**
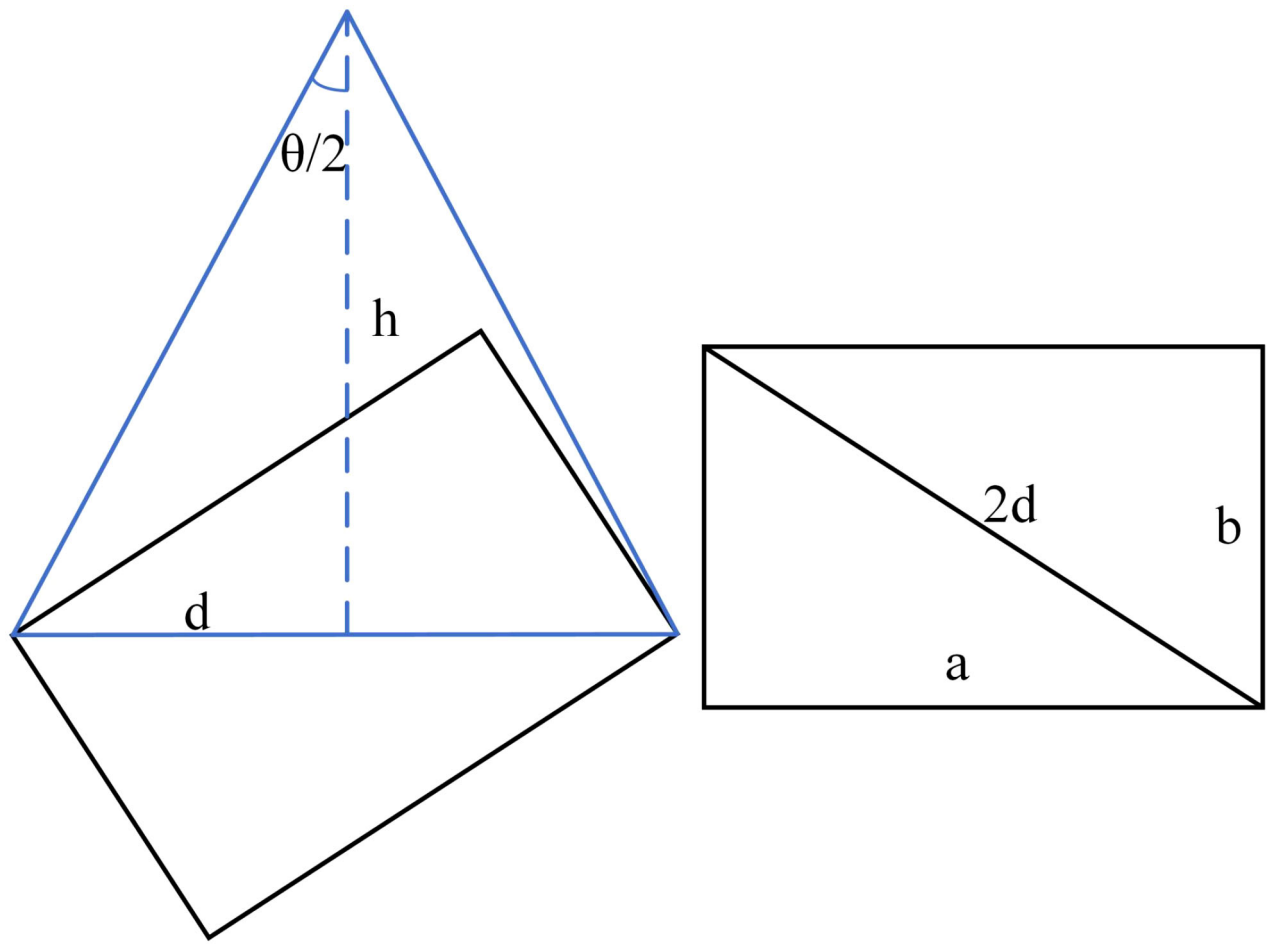
Schematic diagram of the residual film area calculation process.

The residual film coverage area S was calculated by the pixel ratio. Let the size of the aerial image be A×B, and the total number of residual film pixels be p. As shown in **Figure 7**, the aerial photography height is h=5m, and the aerial photography angle is θ=84°. The length of d was calculated to obtain the length of the hypotenuse 2d of the triangle, and the length a and width b of the rectangle were calculated by the Pythagorean theorem. The actual area S1 of the photograph relative to the ground was obtained by multiplying the length by the width (Formula 6). The aspect ratio of the images corresponded to the actual area (**Table 1**).


(6) $$S=\frac{p}{A\times B}\times S1$$

**Table 1:** The aspect ratio of the photo corresponds to the actual area.

Length-to-width ratio	Corresponding area/m^2^
3:2	37.38
4:3	38.89
16:9	34.57

### 2.7 Method for the prediction of residual film content in plough layer

**Figure 8:**
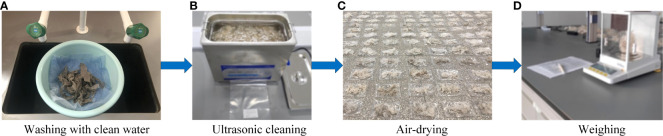
Calculation process of the residual film weight. **(A)** Washing with clean water; **(B)** Ultrasonic cleaning; **(C)** Air-drying; **(D)** Weighing.

(**Figure 8**) The collected residual film was first cleaned with clean water. After that, ultrasonic cleaning was performed, followed by air drying. Finally, the air-dried residual film was weighed, counted, and marked. The residual film area calculation method described was applied to obtain the residual film coverage area S. Regression analysis was carried out with the corresponding residual film mass of the 0-10 cm plough layer, and the obtained mathematical relationship was used to predict the content of residual film in the plough layer. The average value of the residual film content of the plough layer of the five sampling points was taken as the residual film weight of an unit area.

## 3 Results

### 3.1 The identification results of the models

**Figure 9:**
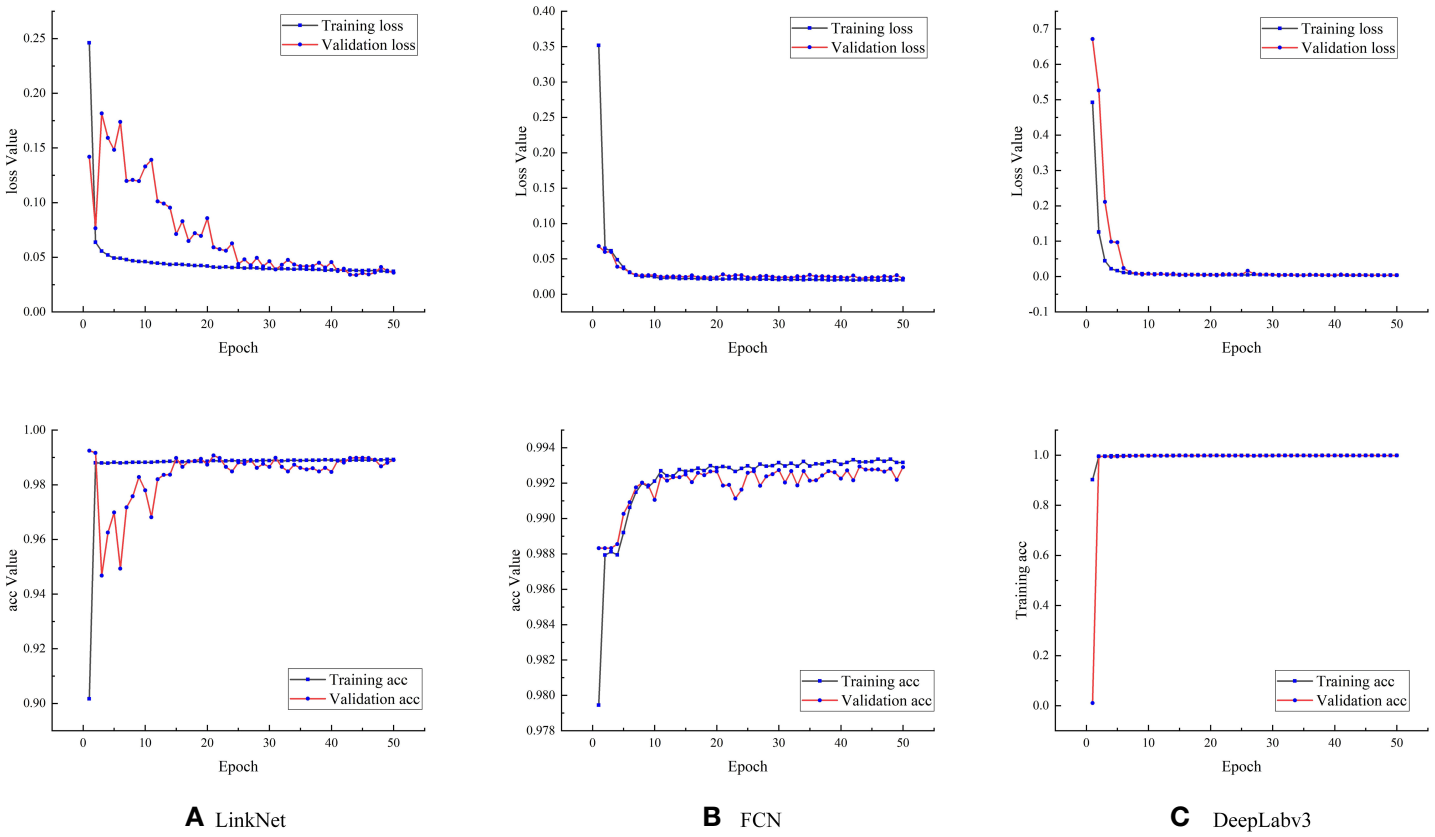
Loss values and accuracies of the three models. **(A)** Loss value and accuracy of the LinkNet model; **(B)** Loss value and accuracy of the FCN model; **(C)** Loss value and accuracy of the Deeplabv3 model.

During the training of the three models (LinkNet, the FCN and DeepLabv3), the loss function decreased rapidly, then converged quickly, and finally stabilized (**Figure 9**). Among the three models, the DeepLabv3 model had the best convergence effect, followed by the FCN model, and the LinkNet model. The accuracy of the three models were relatively high, and during the training process, they quickly reached states of convergence. The DeepLabv3 model had the highest accuracy, followed by the FCN model, and the LinkNet model.

The identification performance of the three models were generally better; among them, the DeepLabv3 model had the best identification performance, with an accuracy of 99.71%, a precision of 85.29%, a recall of 79.38%, an F1 of 79.73%, and a MIOU of 74.62% (**Table 2**). Moreover, the prediction accuracy based on the test set was similar to that based on the training set. It indicates that there is no overfitting.

**Table 2:** Evaluation of different models in segmenting residual from UAV film images.

Model	Accuracy/%	Precision/%	Recall/%	F1- score/%	MIOU/%
LinkNet	98.31	80.11	70.42	71.29	69.77
FCN	99.12	82.51	72.12	73.06	72.12
DeepLabv3	99.71	85.29	79.38	79.73	74.62

**Figure 10:**
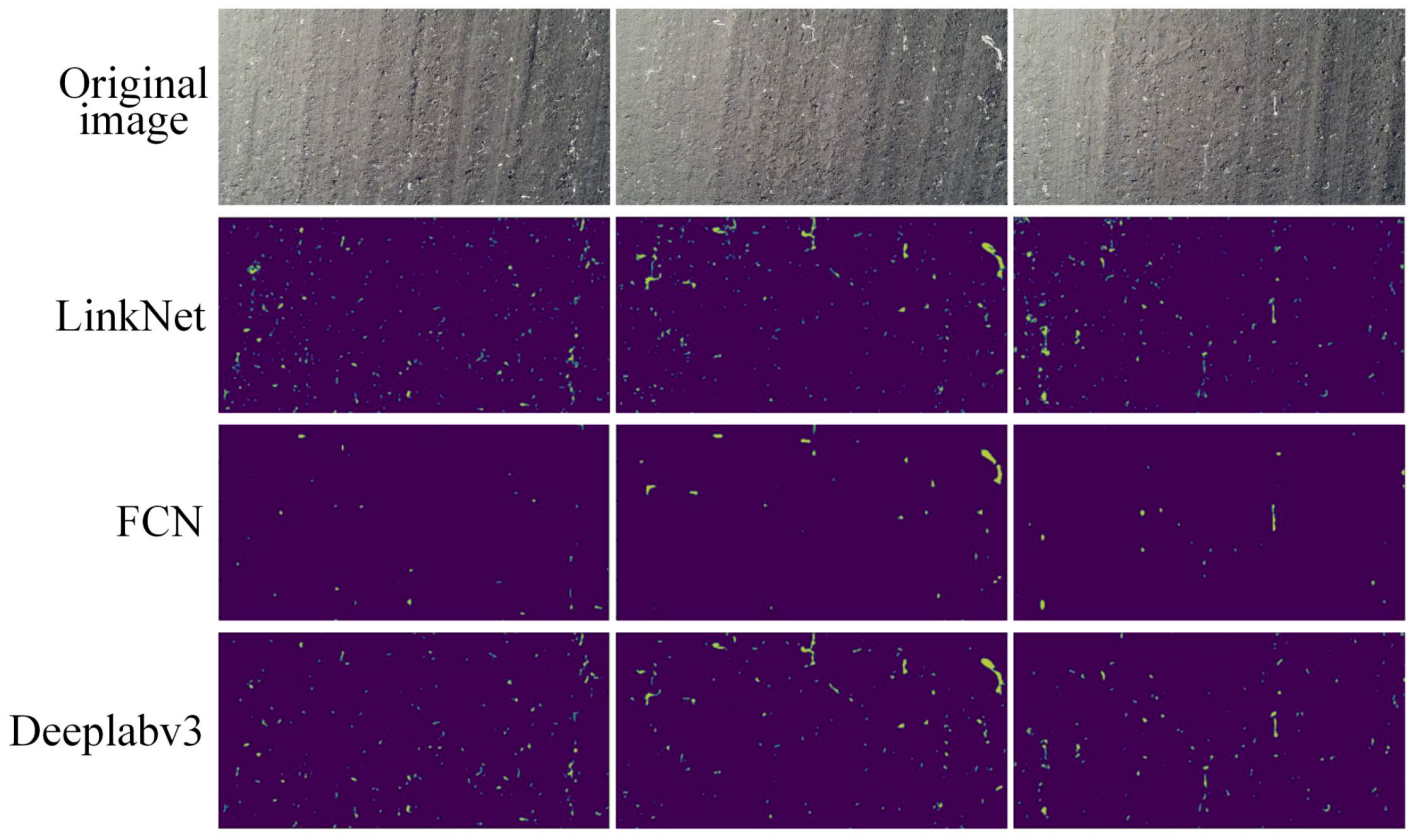
Segmentation performance.

The segmentation results (image size is 5472 × 3078 pixels) predicted by the three models (LinkNet, FCN, and DeepLabv3) (**Figure 10**), showed that the segmentation performance of the three models were generally improved. The DeepLabv3 model had the best segmentation performance, followed by the FCN model, and the LinkNet model. The FCN model failed to identify many small areas of residual film. The LinkNet model had misidentification, and many small soil blocks were misidentified as residual film, resulting in the worst identification performance.

### 3.2 Regression analysis of the residual film content in the plough layer

**Figure 11:**
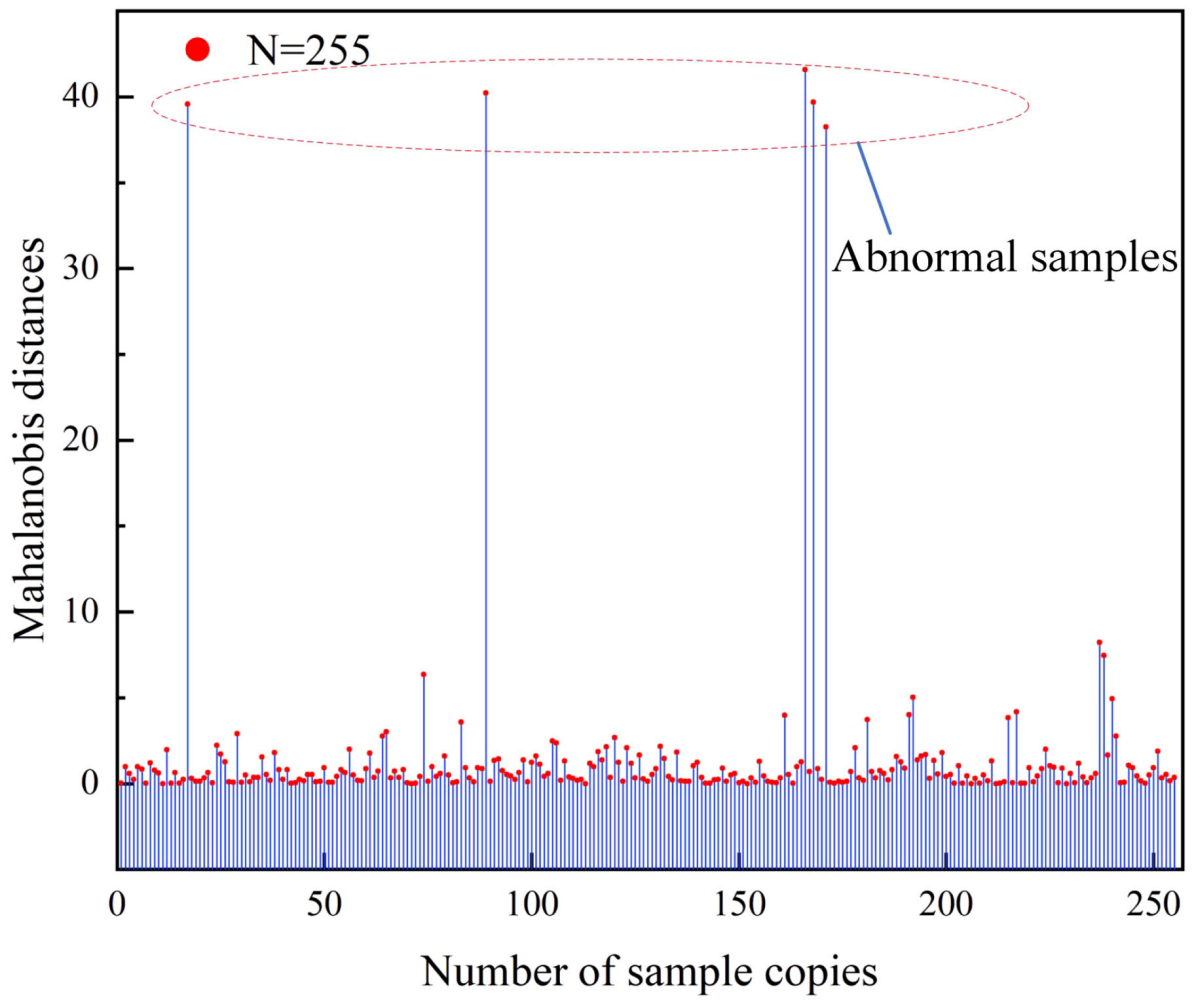
Abnormal sample removal.

Therefore, the DeepLabv3 model was determined as the optimal model for the prediction of the residual film in the plough layer. Then, linear regression analysis was performed on the residual film area and the weight of the residual film in the plough layer calculated by the model. To detect and exclude data outliers, the Mahalanobis distances of 255 sample data were calculated (**Figure 11**). The Mahalanobis distances between the five sets of data and the centre of the dataset were more than three times of the average distance. Therefore, these five sets of data were considered outliers and excluded. The remaining 250 sets of data were used for further analysis and modelling.

**Figure 12:**
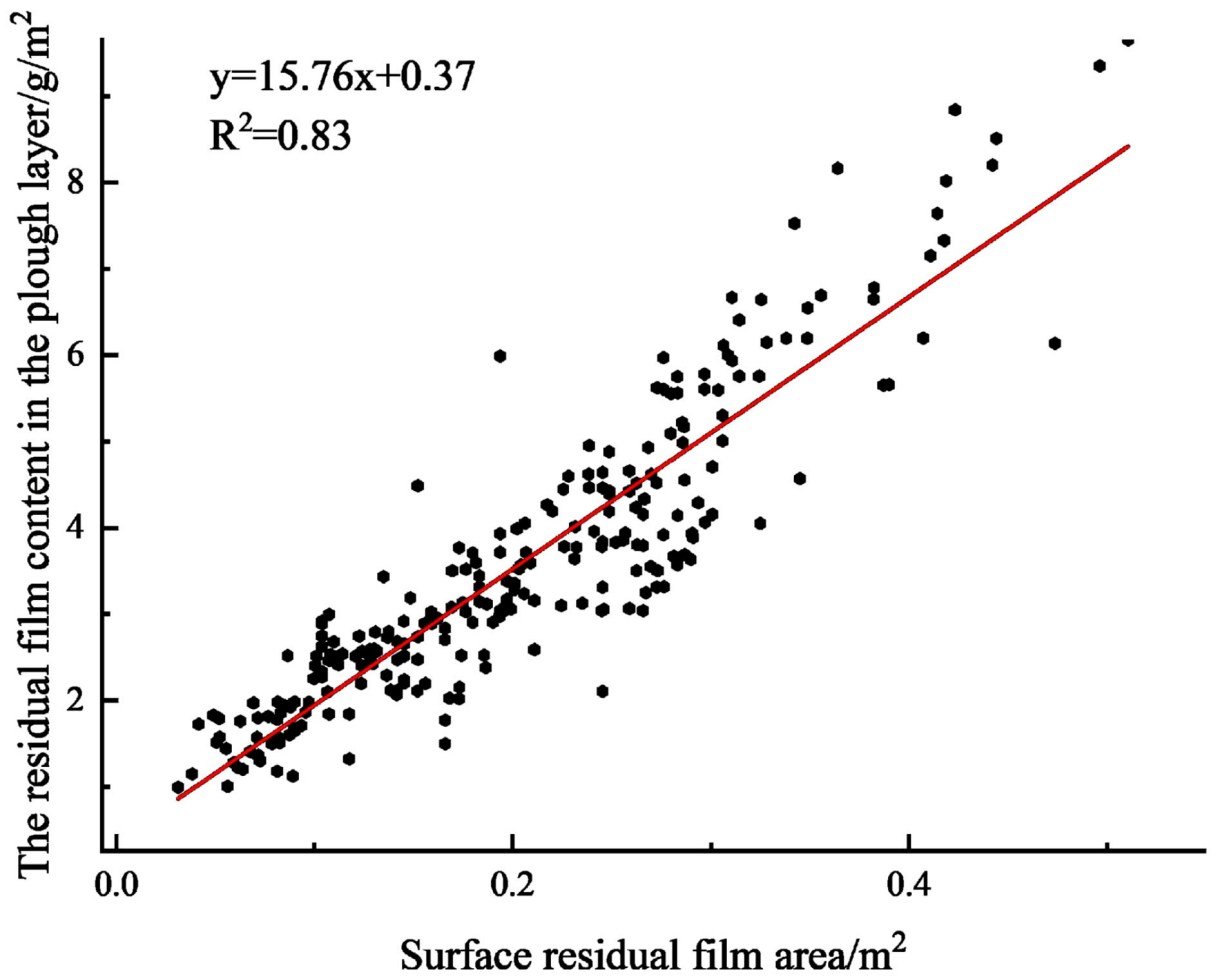
Prediction results for the residual film content in the plough layer.

**Figure 12** shows the analysis results obtained for the 250 sample data. The R^2^ was 0.83, and the root mean square error was 0.48. The mathematical expression y=15.76x+0.37 was obtained for the prediction of the residual film weight in the plough layer, where x is the area of the residual film on soil surface of the cotton field, and y is the weight of the residual film in the plough layer.

### 3.3 Verification of the predicted results

A total of 25 data sets collected from 5 cotton fields were used to verify the prediction model. The drone image were used to calculate the area of the residual film on the soil surface through model identification, and then the residual film content of the plough layer was calculated by the prediction model. The results predicted by the model and the results obtained by manual sampling are shown in **Table 3**. The average relative error of the prediction of the residual film content in the plough layer was 11.06%. It indicates that the proposed method has higher prediction accuracy.

**Table 3:** Comparison of the prediction results regarding the residual film content in the plough layer.

Field number	Average true value/g/m^2^	Average predicted value/g/m^2^	Relative error/%	Average relative error/%
1	4.4647	4.2375	5.08	11.06
2	5.5636	4.8332	13.13	
3	5.9901	4.9221	17.83	
4	6.1139	5.1988	14.97	
5	7.1535	6.8457	4.31	

## 4 Discussion

This paper compared the performance of three deep learning-based semantic segmentation algorithms, LinkNet, FCN, and DeepLabv3, in residual film identification and residual film coverage area prediction. The results showed that the predicted value of the LinkNet model was slightly higher than the real value, and its prediction speed was the fastest. The original intention of this model was to improve the prediction speed. Due to the simple structure and parameter settings of this model, many other things were misidentified as residual films. The parameters of the FCN model were relatively complex, and pretraining weights were introduced, so the model prediction speed was not fast. The model cannot extract the details of the images and does not fully consider the interpixel relationships. Besides, the space regularization used in the segmentation methods based on pixel classification are ignored, resulting in many small residual films not being identified. The DeepLabv3 model had the best segmentation performance, and its segmentation time was between those of the other two models. Chen et al. (2021) used the threshold segmentation method to identify the residual films in cotton fields and found that light intensity had a great influence on it, and its identification accuracy was high. Wu et al. (2020) used the threshold segmentation method to identify residual film in farmland based on colour characteristics and found that the integrity of the residual film was better and that the process of segmenting the residual film was easier compared with other methods. Our study proposed a deep learning method, which can improve the identification accuracy of residual films. However, the dataset of this study needs to be further expanded, and the influence of light intensity on the identification accuracy of the model should be further explored.

In this study, the DeepLabv3 semantic segmentation model was determined as the optimal segmentation model, the area of residual film on soil surface and the residual film weight of the plough layer were analysed by regression analysis, and finally, a regression model was established. The prediction accuracy was high, and the detection speed was greatly improved compared with that of the manual approach. However, the accuracy of the model needs to be further improved, and the influences of different mulching years and different soil qualities on the weight of the residual film in the plough layer should also be considered. Besides, more datasets need to be added to improve the robustness and generalization performance of the model.

## 5 Conclusions

Aiming at the monitoring and evaluation of the residual film content in the cotton field plough layer, a method based on UAV imaging and deep learning was proposed. The conclusions are drawn as follows.

(1) Compared with the LinkNet, FCN and DeepLabv3 models, the DeepLabv3 semantic segmentation model had the best performance, with accuracy, precision, recall, F1-score, and MIOU values of 99.71%, 85.29%, 79.38%, 79.73%, and 74.62%, respectively.

(2) A method for predicting residual film contents in the cotton field plough layer was proposed. The regression model was established by fitting the area of the residual film on soil surface and the weight of the corresponding residual film in the plough layer. The R^2^ of the regression model was 0.83, and the root mean square error was 0.48.

(3) The accuracy of the proposed method for predicting the residual film contents in the cotton field plough layer was verified. The results showed that the proposed method achieved a faster detection and a higher prediction accuracy, and the average relative error was 11.06%. This study makes up for the deficiency that the current monitoring methods can only evaluate the content of residual film on soil surface and provides an effective method for monitoring and evaluating the residual film pollution in the plough layer.

## Data availability statement

The original contributions presented in the study are included in the article/supplementary material. Further inquiries can be directed to the corresponding authors.

## Author contributions

FQ and ZZ who contributed equally to this work share the first authorship. They collected and analysed the data and wrote the manuscript under the supervision of RZ and YL. JY and HW assisted in collecting and analyzing the data. All authors reviewed and revised the manuscript.

## Funding

The authors gratefully acknowledge the financial support provided by the National Natural Science Foundation of China (32060412), the High-level Talents Research Initiation Project of Shihezi University (CJXZ202104), the Earmarked Fund for China Agriculture Research System (CARS-15-17) and the Graduate Education Innovation Project of Xinjiang Autonomous Region (XJ2022G082). 

## Acknowledgments

The authors would like to thank Jie Huang and Hao Pan for their assistance with the experiment.

## Conflict of interest

The authors declare that the research was conducted in the absence of any commercial or financial relationships that could be construed as a potential conflict of interest.

## Publisher’s note

All claims expressed in this article are solely those of the authors and do not necessarily represent those of their affiliated organizations, or those of the publisher, the editors and the reviewers. Any product that may be evaluated in this article, or claim that may be made by its manufacturer, is not guaranteed or endorsed by the publisher.
